# Echocardiographic Global Longitudinal Strain and Myocardial Fibrosis in Patients with Left Ventricular Hypertrophy and Hypertrophic Cardiomyopathy

**DOI:** 10.3390/biomedicines14061278

**Published:** 2026-06-04

**Authors:** Monika Matla-Hajzyk, Mariusz Balys, Aleksander Olejnik, Patrycja Brzoska, Maciej Haberka

**Affiliations:** Department of Cardiology, School of Health Sciences, Medical University of Silesia, 40-055 Katowice, Polandpatrycja.brzoska1@poczta.onet.eu (P.B.);

**Keywords:** hypertrophic cardiomyopathy, global longitudinal strain, speckle-tracking echocardiography, cardiac magnetic resonance, late gadolinium enhancement, myocardial fibrosis

## Abstract

**Background:** Myocardial fibrosis is an important pathological feature of hypertrophic cardiomyopathy (HCM) and is associated with ventricular arrhythmias, disease progression, and adverse clinical outcomes. Cardiovascular magnetic resonance (CMR) with late gadolinium enhancement (LGE) is the reference non-invasive technique for myocardial fibrosis assessment; however, its availability may be limited. Global longitudinal strain (GLS) derived from transthoracic echocardiography (TTE) has emerged as a sensitive marker of myocardial dysfunction and may provide complementary information regarding myocardial involvement. **Aim:** The aim of our study was to evaluate the diagnostic value of transthoracic echocardiography (TTE) with 2D global longitudinal strain (GLS) to detect the degree of myocardial fibrosis (LGE) in patients with LV hypertrophy (LVH). **Methods:** A total of 95 consecutive patients referred for cardiovascular magnetic resonance (CMR) because of suspected hypertrophic cardiomyopathy or left ventricular hypertrophy were screened for eligibility. After applying exclusion criteria and excluding patients with alternative diagnoses or inadequate image quality, 83 patients were included in the final analysis. All the participants underwent both CMR and transthoracic echocardiography with GLS assessment. **Results:** The final study population included 83 patients (57.5 ± 13 years; 66% males). CMR confirmed HCM in 58 (70%) patients, including 23 with left ventricular outflow tract obstruction (LVOTO). The remaining patients demonstrated varying degrees of left ventricular hypertrophy that did not fulfill established diagnostic criteria for hypertrophic cardiomyopathy. Cardiovascular magnetic resonance studies (58 cases; 69%) showed a non-ischemic LGE in LV (23% of segments with LGE). GLS in patients with LGE was significantly lower than those without LGE (−13.9 ± 3.6 vs. −15.9 ± 2.7%, *p* = 0.01). The mean GLS was −14.52 ± 3.5% and showed a moderate positive correlation with the extent of myocardial fibrosis (LGE%LV; r = 0.45, *p* < 0.01). This relationship remained significant in multivariable regression analysis (standardized coefficient = 0.683; *p* < 0.05). Moreover, the transthoracic echocardiography GLS showed a significant association for LV LGE (−14.3%; AUC 0.658; *p* = 0.01, sensitivity 39%, specificity 90%) with a better diagnostic performance for LGE in more than four LV segments (−12.1%; AUC 0.867; *p* < 0.001, sensitivity 72%, specificity 87%). **Conclusions:** GLS was independently associated with myocardial fibrotic burden assessed by CMR. Although it cannot replace CMR for tissue characterization, GLS may provide adjunctive information and may help identify patients with greater fibrotic burden. Prospective studies are needed to validate its clinical utility.

## 1. Introduction

Hypertrophic cardiomyopathy (HCM) is a hereditary cardiovascular disease affecting one in 500 people, with heart failure and sudden cardiac death being the leading causes of death [1]. Myocardial fibrosis is an important histopathological feature of HCM, resulting in myocardial remodeling, electrical inhomogeneity, and both myocardial systolic and diastolic dysfunction [1,2]. In the past, myocardial biopsy was the gold standard for detecting myocardial fibrosis [3]. However, because of its invasive nature, limited sampling sites and advanced cardiac imaging, cardiovascular magnetic resonance (CMR) has become the clinical standard for myocardial tissue characterization [4,5]. CMR late gadolinium enhancement (LGE) is a well-evidenced non-invasive method for detecting myocardial fibrosis [6]. In contemporary practice, a multimodality imaging approach integrating echocardiography and CMR plays a central role in the diagnosis, risk stratification, and management of patients with hypertrophic cardiomyopathy [7].

Transthoracic echocardiography (TTE) is the most common and the first imaging modality for patients with suspected HCM [4,5]. However, it has some limitations in selected cases and routine TTE does not allow for the assessment of myocardial injury, except for the left ventricular ejection fraction (LVEF). Speckle-tracking echocardiography (STE) could provide more sensitive parameters for the early identification of myocardial injury, which may improve patient risk assessment. Among various STE parameters, left ventricular (LV) global longitudinal strain (GLS) is the most robust and validated parameter of LV damage recommended in the European Society of Cardiology (ESC) guidelines [5].

Therefore, this study aimed to assess the diagnostic value of TTE GLS in relation to myocardial fibrosis evaluated by CMR in patients with left ventricular hypertrophy (LVH).

## 2. Material and Methods

Patients were scheduled for CMR due to HCM suspected in TTE performed in an outpatient’s clinic. All the enrolled patients underwent both TTE and CMR examinations, performed by a study team with long-standing experience in cardiac imaging.

The main inclusion criteria were LVH and/or HCM suspected in TTE by the referring cardiologist in an outpatient center. The main exclusion criteria were as follows: secondary cause of LVH (including cardiac amyloidosis, Anderson–Fabry disease), history of myocardial infarction, history of an implantable cardiac device (pacemaker, implantable cardioverter or cardiac resynchronization therapy), at least moderate arterial hypertension, chronic kidney disease (eGFR < 60 mL/min/1.73 m^2^), uncontrolled diabetes, heart valve stenosis of any degree, significant native valve regurgitation, poor image quality for speckle-tracking analysis, and contraindication for contrast-enhanced CMR. The main clinical indication for CMR in all the participants was HCM in TTE. However, CMR is the reference imaging modality for the evaluation of LV myocardium. It confirms the primary diagnosis (HCM) in most cases, but it also shows lower degrees of LVH, which are mostly the preclinical stages of future HCM. Given that further clinical decisions are strictly based on the confirmed diagnosis of HCM, the study group was divided into two subgroups based on CMR results: HCM (LV segment ≥15 mm) and non-HCM (LV segments <15 mm).

All the participants underwent comprehensive clinical assessment including medical history and standard evaluation for potential causes of left ventricular hypertrophy. Individuals with features suggestive of physiological hypertrophy (athlete’s heart), including a history of intensive competitive athletic training or clinical characteristics inconsistent with pathological hypertrophy, were not identified in the study population. Family history and clinical context were considered as part of the routine diagnostic assessment.

Written informed consent was obtained from all the patients included into the study. The study protocol conformed to the ethical guidelines of the 1975 Declaration of Helsinki. The study protocol was approved by the Medical University of Silesia Bioethics Committee (KNW/0022/KB1/77/17, PCN/CBN/022/KB1/77/I/17/21).

This was a prospective, non-interventional study, and it was supported by a statutory research grant for the Medical University of Silesia (BNW-1-164/K/3/K).

### 2.1. Transthoracic Echocardiography

Comprehensive 2D and Doppler echocardiography was performed using a commercially available ultrasound machine and transducer (Vivid E95, GE, Healthcare, Chicago, IL, USA), and images were analyzed using EchoPAC software (version 203). Echocardiographic parameters were obtained according to the American Society of Echocardiography (ASE) guidelines [8,9,10].

All the views at three apical views (apical 4-chamber, 2-chamber, and long-axis) encompassing the entire left ventricle were acquired. Pulsed-wave Doppler of left ventricular (LV) inflow and outflow, as well as tissue Doppler imaging of the mitral annulus, were performed in accordance with the ASE recommendations.

The echocardiographic parameters included standard measurements and GLS using two-dimensional STE. The two-dimensional speckle-tracking analysis was performed using EchoPAC software with the Automated Functional Imaging module. Longitudinal strain was measured by automatic tracing of the endocardial border in the three apical views. The quality of tracking was verified visually, and if tracking was deemed suboptimal, manual adjustment of the endocardial border was performed. All echocardiographic studies were performed by two experienced echocardiographers. GLS analysis showed excellent reproducibility with low intraobserver and interobserver variability (ICC 0.95 and 0.92, respectively).

**Figure 1:**
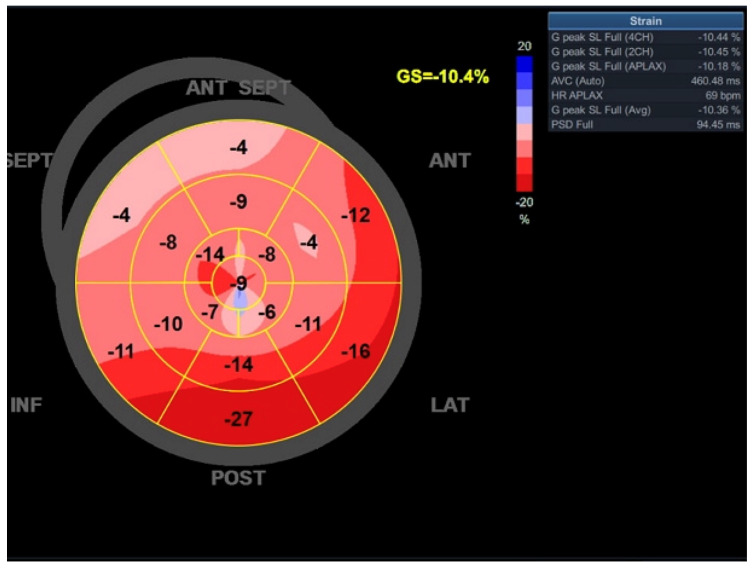
Impaired left ventricular global longitudinal strain assessed by speckle-tracking echocardiography in a patient with hypertrophic cardiomyopathy.

**Figure 2:**
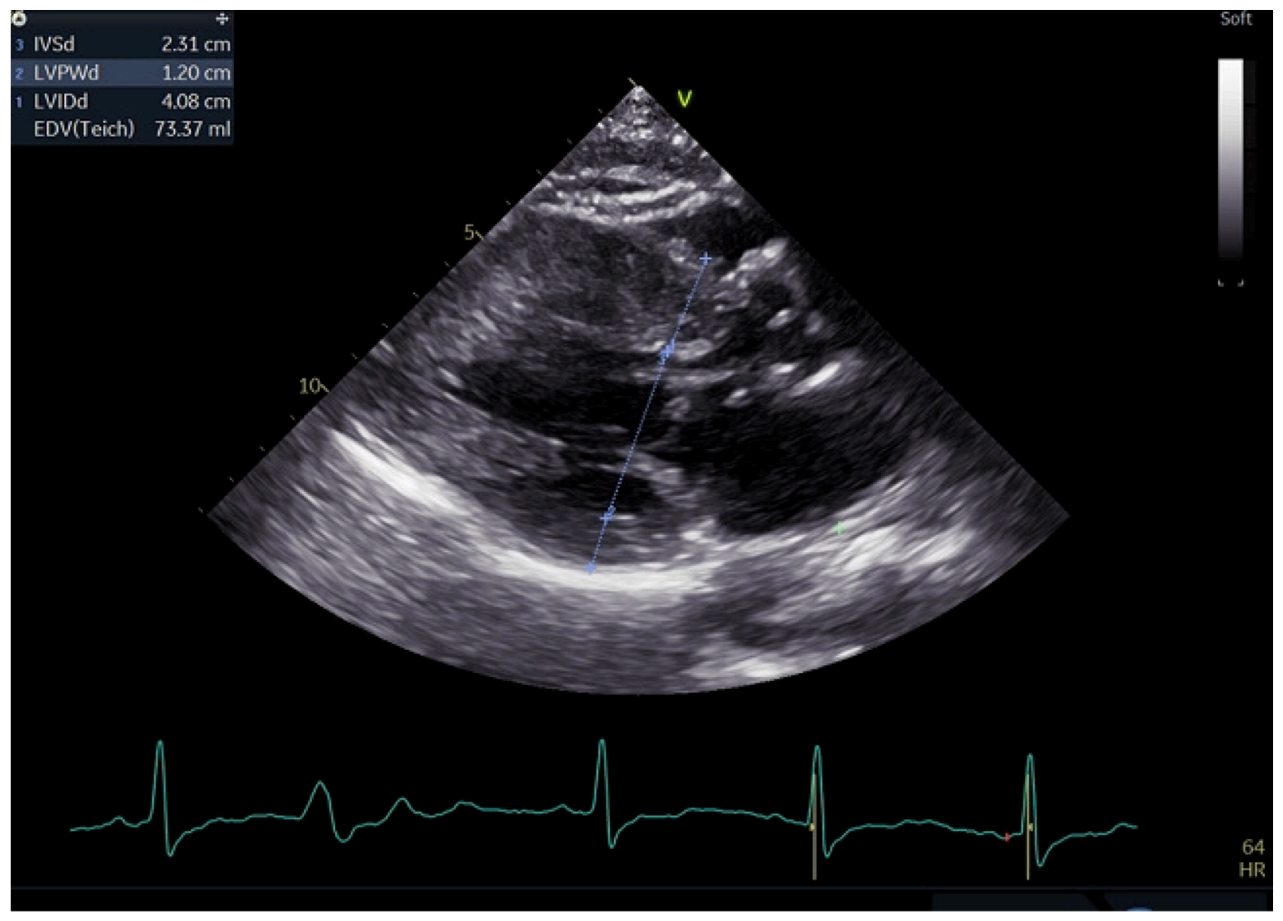
Parasternal long-axis echocardiographic view of the left ventricle in a patient with hypertrophic cardiomyopathy.

Representative echocardiographic findings, including impaired GLS, are presented in Figure 1 and Figure 2.

### 2.2. Cardiac Magnetic Resonance

All the CMR images were obtained blinded to the patient’s echocardiographic data on the 1.5 T system (GE Healthcare, Waukesha, WI, USA) with a dedicated phased-array cardiac coil. All CMR examinations were ECG-gated using a comprehensive clinical protocol in line with current guidelines [11,12]. The CMR protocol was described in our previous paper [13].

In brief, the following sequences were performed: multi-planar SSFP acquisitions for functional sequences in short- and long-axis, T2-weighted edema imaging with fat saturation sequences, and LGE sequences for fibrosis evaluation performed 10–15 min after contrast infusion (0.1 mmol/kg of body weight of gadobutrol, Gadovist 1.0, Bayer Inc., Mississauga, ON, Canada). All the LGE acquisitions were based on the same planes as the short- and long-axis SSFP images: transverse short-axis LV planes and longitudinal LV planes.

The CMR scans were obtained and analyzed by an experienced operator with a long-standing practice and the European Association of Cardiovascular Imaging certificate. Cardiac volumes, mass, and function (left and right ventricle end-diastolic and end-systolic volumes, ejection fraction (EF)) were analyzed using a dedicated commercial software (CVI42, version number 6.3, Circle Cardiovascular Imaging Inc., Calgary, AB, Canada). All volumes and LV mass were indexed to body surface area (BSA) and were interpreted using gender- and age-adjusted reference values provided by the European Association of Cardiovascular Imaging guidelines [14,15]. The LV myocardium was segmented according to the American Heart Association 17-segment model [16].

**Figure 3:**
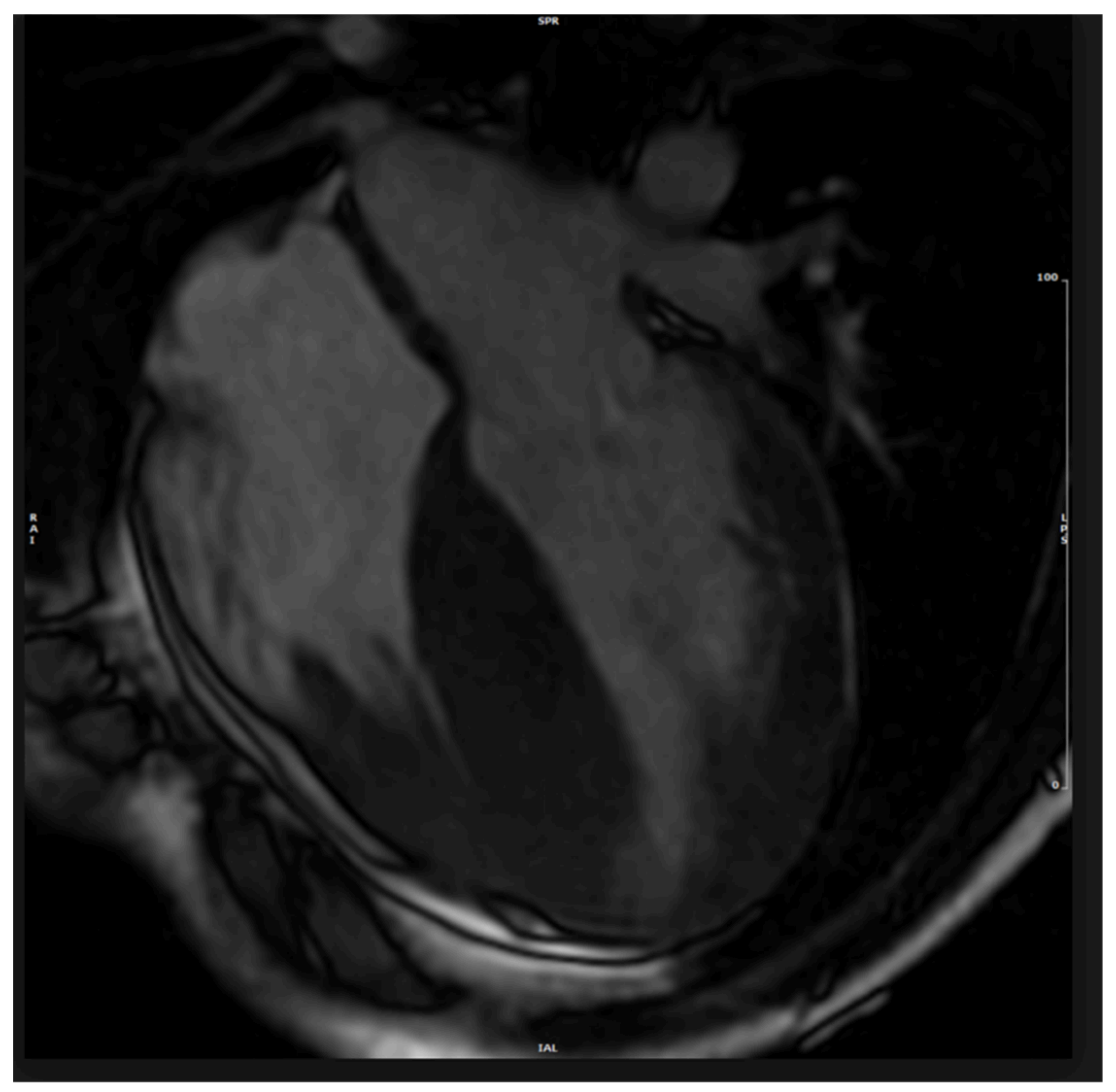
Cardiovascular magnetic resonance: a long-axis SSFP sequence in 4-chamber view in a patient with hypertrophic cardiomyopathy.

**Figure 4:**
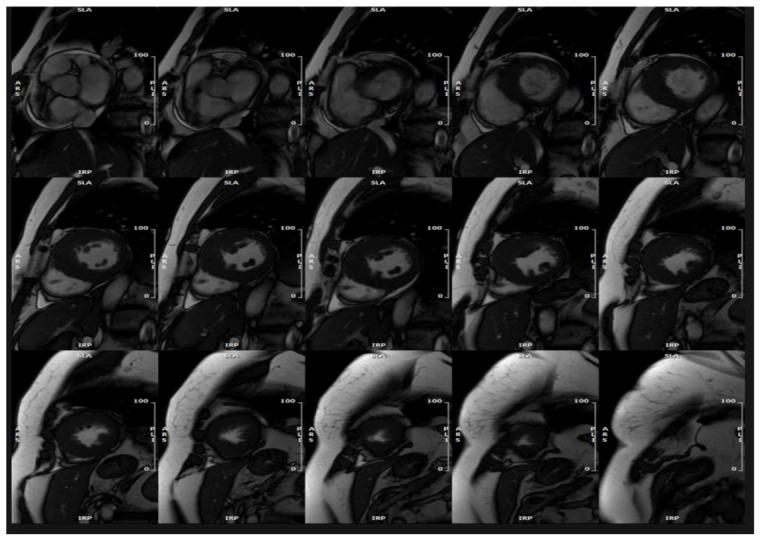
Cardiovascular magnetic resonance: a short-axis SSFP sequence in left ventricle view in a patient with hypertrophic cardiomyopathy.

**Figure 5:**
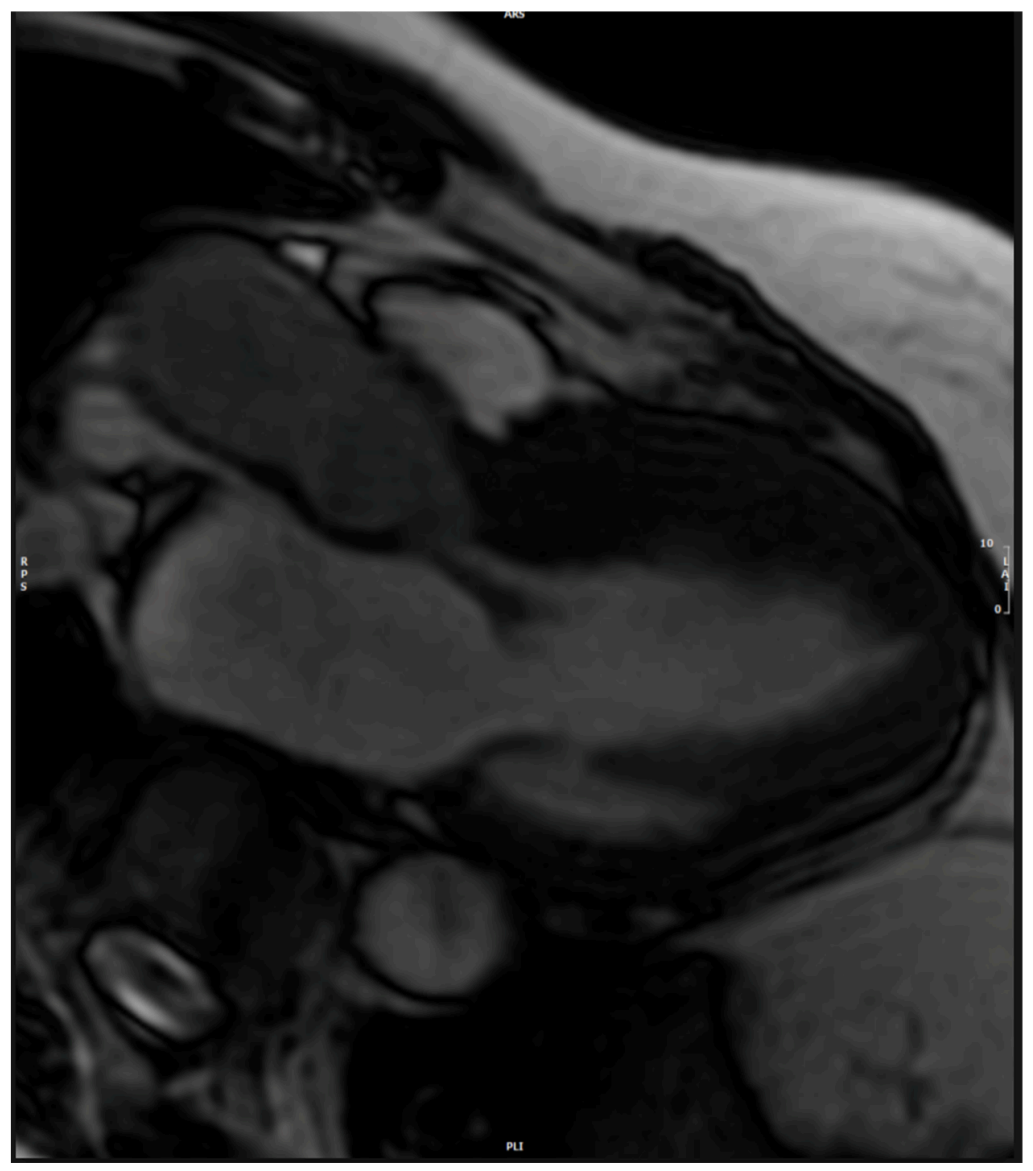
Cardiovascular magnetic resonance: a long-axis SSFP sequence in 3-chamber view in a patient with hypertrophic cardiomyopathy.

**Figure 6:**
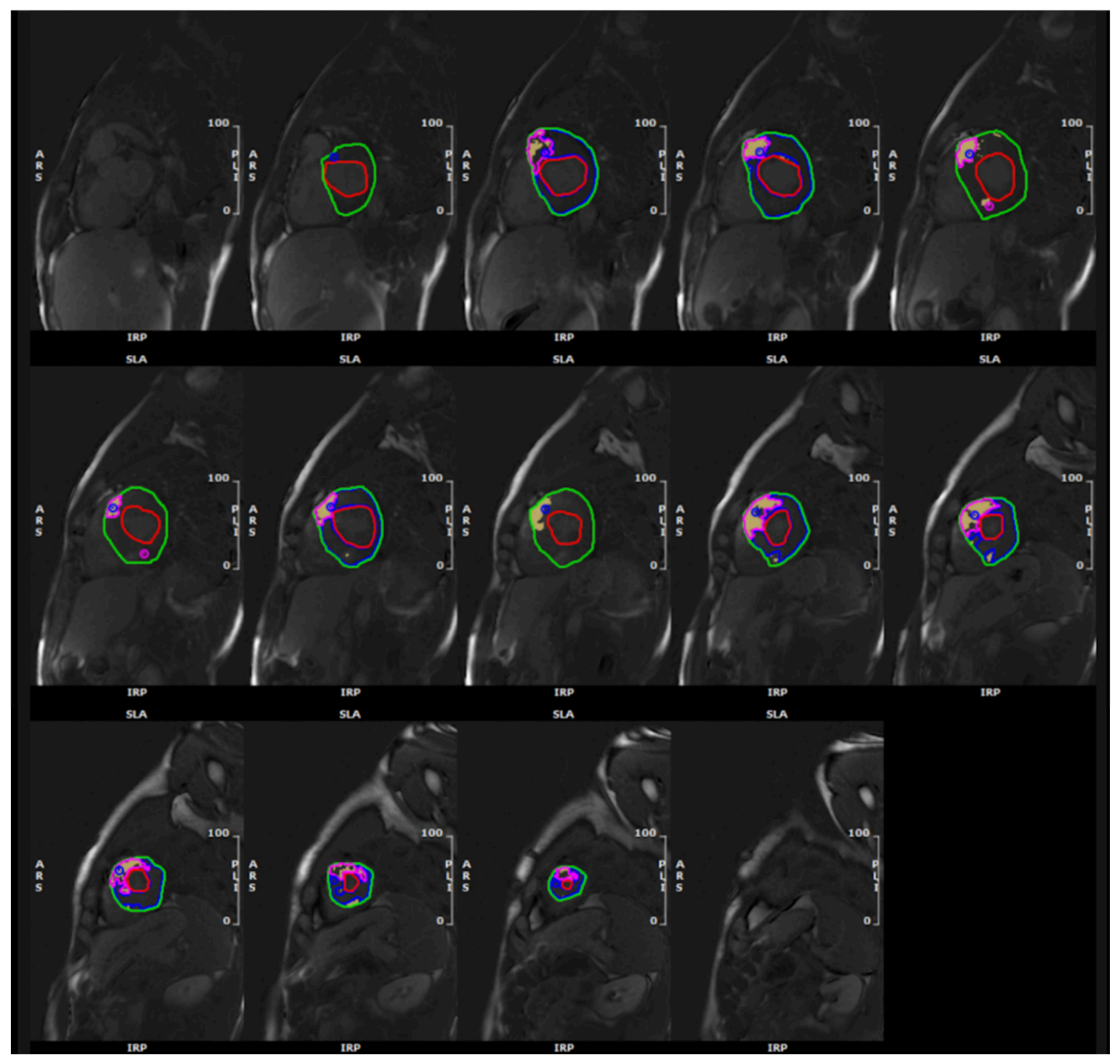
Cardiovascular magnetic resonance: a short-axis late gadolinium enhancement sequence in left ventricle view in a patient with hypertrophic cardiomyopathy.

Myocarditis-like injury was defined as non-ischemic mid-wall and/or subepicardial LGE [11,12]. All the segments were evaluated for the presence and the severity of LGE by the semiquantitative method, which was calculated as the ratio of LGE area to the segment area. Afterward, the values (%) for each segment were summed up in total LGE values expressed as a ratio (%) and mass (g). Representative examples of CMR findings in patients with HCM are shown in Figure 3, Figure 4, Figure 5 and Figure 6.

### 2.3. Statistical Analysis

All continuous variables were assessed for normality using the Shapiro–Wilk test. Normally distributed data are expressed as the mean ± standard deviation (SD) and compared using Student’s *t*-test. The median and interquartile range (IQR) were used for non-normally distributed data, and the Mann–Whitney U-test was performed for comparison. A receiver operating characteristic (ROC) curve was used to calculate the area under the curve (AUC) to explore the predictive value of each parameter in detecting myocardial fibrosis. In addition, the optimal cut-off value for specificity or sensitivity was calculated by maximizing the Youden index. The De Long test was used to compare the AUC values. To detect independent risk factors for predicting significant myocardial fibrosis, variables with significant *p*-values in univariate analysis were further subjected to multivariable linear regression analysis. To avoid model overfitting, the number of variables included in the multivariable model was limited relative to the sample size, and only variables with statistical significance in univariate analysis and clinical relevance were entered into the final model. Pearson’s correlation coefficient (r) was used to examine the association between GLS and the number of positive LGE segments. A two-tailed *p* < 0.05 was considered significant in all tests. Multicollinearity was assessed by examining pairwise correlations between independent variables. A correlation coefficient <0.7 was considered indicative of acceptable collinearity. All statistical analyses were performed using MedCalc Software Ltd., Ostend, Belgium, ver. 23.5.

## 3. Results

### 3.1. Study Population and Clinical Characteristics

**Scheme 1:**
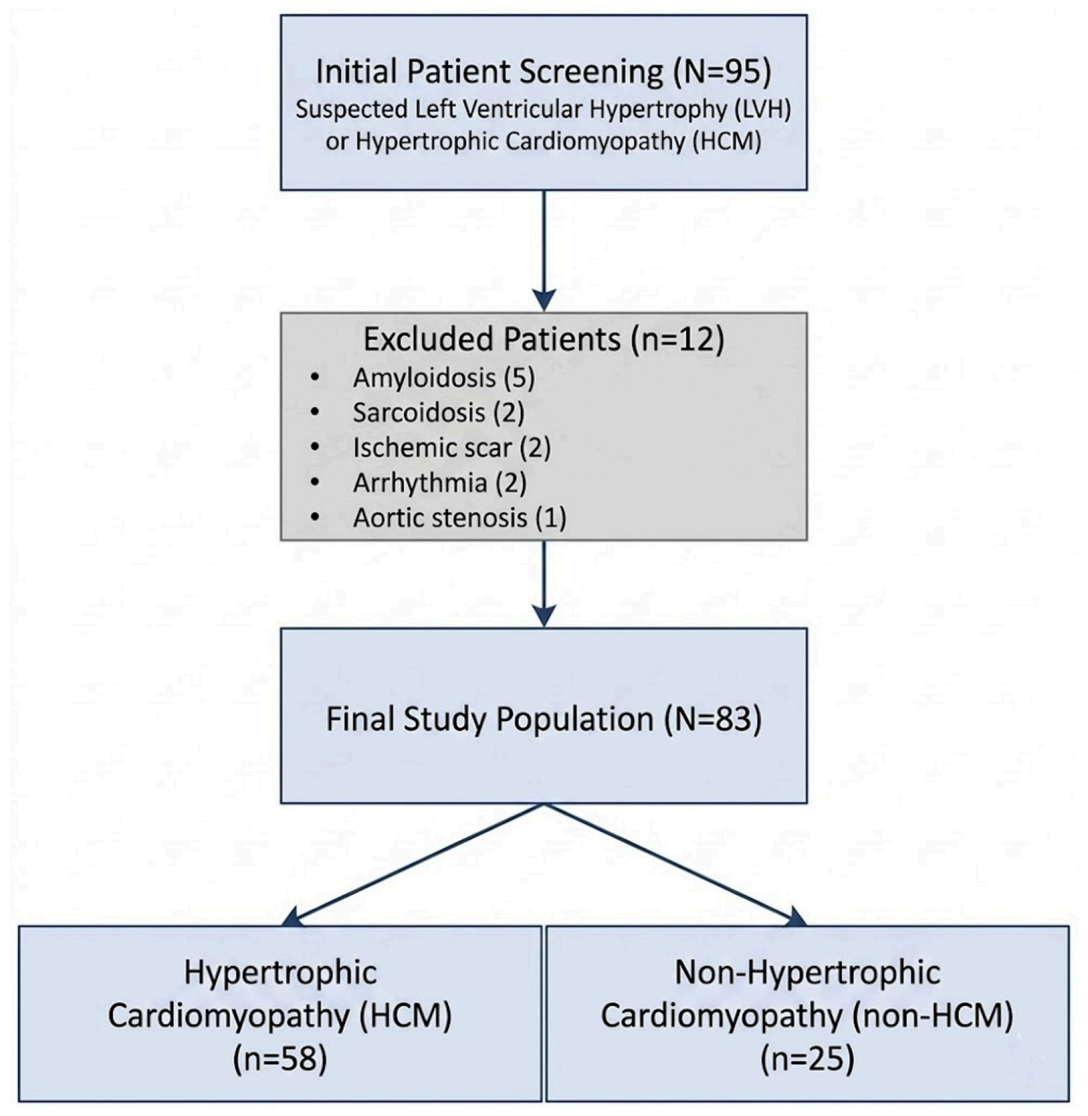
The distribution of patients in the study group. Abbreviations: LVH—left ventricular hypertrophy; HCM—hypertrophic cardiomyopathy.

All 95 consecutive patients with suspected LVH and HCM were enrolled in the study. The median time between TTE and CMR was 14 (8–18) days. Finally, 12 patients were excluded from the analysis due to cardiac amyloidosis (*n* = 5), cardiac sarcoidosis (*n* = 2), or myocardial ischemic scar (*n* = 2) found in CMR. Moreover, another two patients suffered from uncontrolled arrhythmia (poor image quality), and one patient was found to have a significant aortic valve stenosis (Scheme 1). The final study group included 83 individuals (66% males) with an average age of 57.5 ± 13.3. Most patients had preserved LVEF (96%) with limited symptoms (57% in NYHA class I). Blood pressure values remained within the normal range during the clinical visit associated with echocardiographic examination and immediately before CMR acquisition. Heart rate was stable and remained below 80 beats/min during both the TTE and CMR examinations. These measures were considered to minimize the influence of acute hemodynamic conditions on strain assessment and imaging-derived parameters. The clinical characteristics of the study group and CMR parameters are presented in Table 1.

### 3.2. HCM and Non-HCM Patients

CMR confirmed HCM in 58 patients, including 23 individuals with left ventricular outflow tract obstruction. Meanwhile, the remaining patients demonstrated varying degrees of LV hypertrophy but did not meet established diagnostic criteria for HCM. Only three patients were found to have an LV systolic dysfunction (LVEF < 50%), and 77% of the patients had LV hyperkinesis, typical for HCM (LVEF > 60%). Regional wall motion abnormalities were found in 19 (22%) patients within the most hypertrophied segments.

Most individuals in both subgroups were men. The patients in the non-HCM subgroup were younger and showed mainly limited symptoms (NYHA class I). As expected, CMR showed more advanced hypertrophy and larger LV mass in the HCM patients compared to the non-HCM subgroup. Except for a higher rate of arterial hypertension in the HCM group, there were no differences in comorbidities (Table 2).

**Figure 7:**
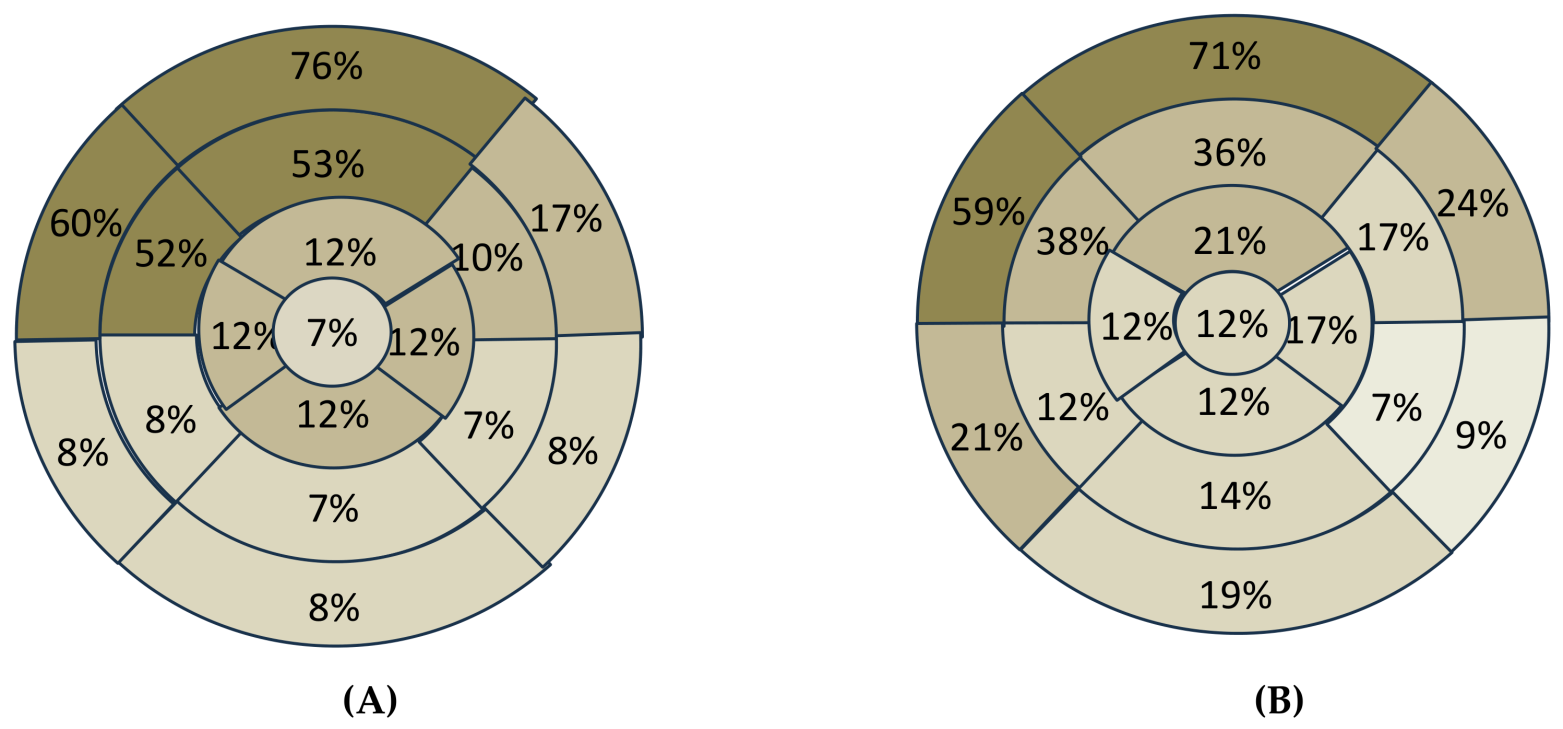
Bull’s eye plots showing left ventricle segments with the location and distribution of hypertrophy (≥15 mm) (**A**) and late gadolinium enhancement (LGE) (**B**) according to the 17-segment AHA (American Heart Association 17-segment model).

There were various patterns of LVH in the patients with HCM. Most cases were found to have hypertrophy within the basal and/or mid segments within the anterior and anteroseptal wall. Figure 7A shows left ventricle segments, according to the 17-segment American Heart Association model, with location and distribution of hypertrophy (≥15 mm).

Most of the CMR studies (58 cases; 69%) showed LGE in LV myocardium. The rate of LV segments with LGE was 23% and the median number of LV segments with LGE was 2 (0–4). Among the 58 patients with LGE, 229 LV segments were found to have LGE, and 194 (84%) revealed an intramyocardial pattern. The median total LGE in LV was 2.95% (0–5.9%), with 14 cases found to have a significant LGE (>10% of LV mass). The basal anterior and anteroseptal segments were the most frequent locations of myocardial fibrosis (LGE)—Figure 7B.

### 3.3. TTE with GLS and Association with LV Myocardial Fibrosis

All the study patients were scheduled for both TTE and CMR, which were assessed blinded to the clinical data and by independent observers. The conclusions for HCM of both modalities were concordant in 82% of the cases. The HCM was overdiagnosed in 10 patients and underdiagnosed in five cases compared to CMR, which was mainly due to borderline hypertrophy.

**Figure 8:**
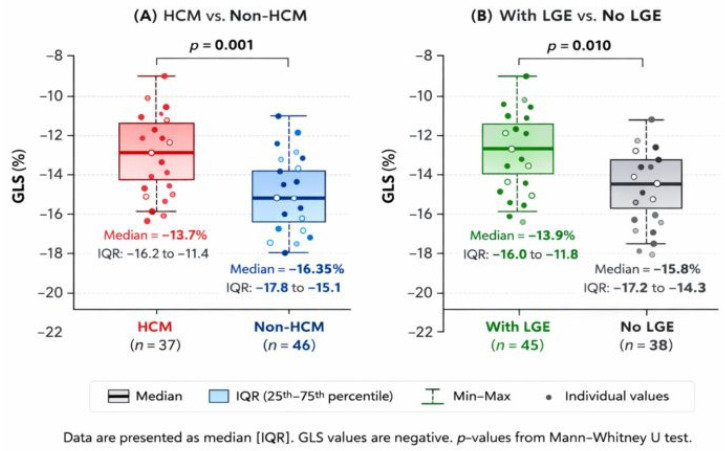
Comparison of global longitudinal strain (GLS) in the study group—in echocardiography (**A**) and magnetic resonance (**B**) modalities. (**A**) **A** box-and-whiskers plot; the central box represents the values between the 25th and 75th percentiles. The middle line represents the mean. The horizontal line extends from the minimum to the maximum value. (**B**) A box-and-whiskers plot; the central box represents the values between the 25th and 75th percentiles. The middle line represents the mean. The horizontal line extends from the minimum to the maximum value.

The mean echo GLS in the study group was −14.52 ± 3.5%. The patients with HCM showed significantly impaired GLS compared to the non-HCM individuals (−13.7 ± 3.5% vs. −16.35 ± 2.9%; *p* = 0.001; Figure 8A). Moreover, GLS was significantly impaired in patients with LGE compared to those without myocardial fibrosis (*p* = 0.01; Figure 8B).

The mean GLS was also associated with the number of segments with myocardial fibrosis (LGE). It was significantly lower in the subgroup with LGE in 0–2 segments compared to the subgroup with LGE in more than two segments (−15.7 ± 3.3 vs. −12.6 ± 3; *p* = 0.01) and between the subgroups with LGE in 0–4 segments vs. the patients with LGE > 4 segments (−15.1 ± 3.2 vs. −10.7 ± 2.7; *p* = 0.0001). The severity of LGE in LV (LGE%LV) was associated with GLS (r = 0.42; *p* = 0.0001) and CMR LV mass (r = 0.35; *p* = 0.001) or LVEF (r = −0.3; *p* = 0.007). The Table 3 shows multivariate linear regression model analysis of echocardiographic parameters and the severity of myocardial fibrosis (LGE) in the patients with LVH.

### 3.4. TTE with GLS in Association for the Severity of Myocardial Fibrosis

**Figure 9:**
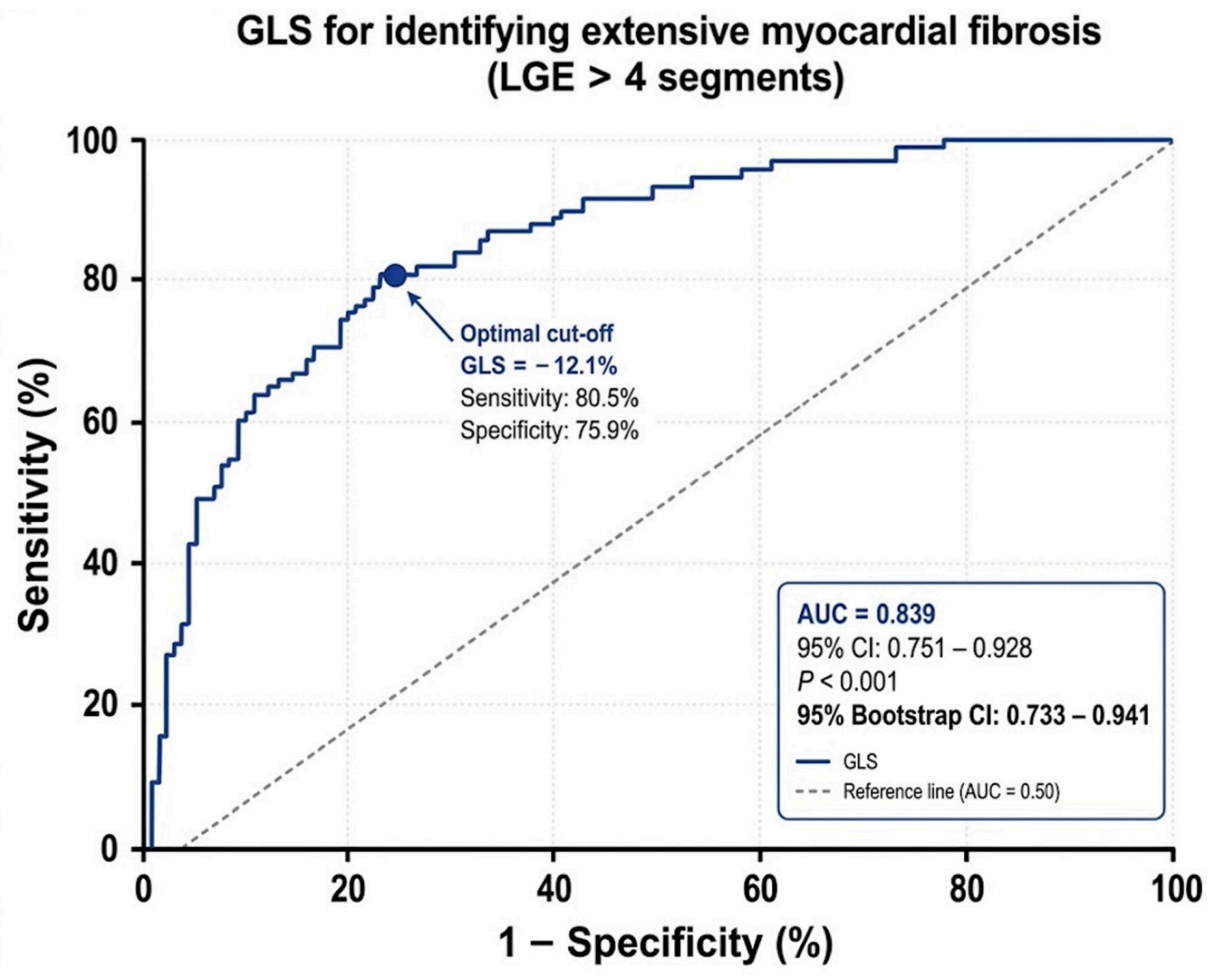
Receiver operating characteristic (ROC) curve for GLS in predicting severe myocardial fibrosis (LGE > 4 segments). AUC = 0.867 (95%), *p* < 0.001.

Table 4 shows the ROC analysis for different indices as associated parameters of left ventricular fibrosis. Figure 9 provides the receiver operating characteristic curve parameters of the TTE parameters as associated parameters of LV fibrosis (AUC 0.867).

All the echocardiographic parameters were used in the ROC curve analysis to predict myocardial fibrosis in the LV. However, only age, E/E’, and GLS were the significant predictors of myocardial fibrosis (LV LGE). Moreover, GLS was the only TTE parameter that showed a significant predictive value for a moderate (>2 LV segments) or severe (>4 LV segments) myocardial fibrosis (Table 4). The threshold of >4 LGE-positive segments was selected to identify patients with more advanced myocardial fibrosis, based on the distribution of LGE in the study population and in line with prior segment-based approaches used in the literature [17,18]. The AUC values for age, E/E’, and GLS were similar, with the highest sensitivity for age >55 and the best specificity for GLS (<−14.3). The AUC values of GLS in prediction for LGE, LGE > 2 segments and LGE > 4 segments were compared and showed no significant differences (*p* = 0.7). However, GLS showed the highest specificity in the prediction of severe myocardial fibrosis (LGE > 4 LV segments) (Figure 9 and graphical abstract).

### 3.5. TTE with GLS and Association with LV Myocardial Fibrosis

#### GLS and LV Myocardial Fibrosis in Patients with HCM

Among patients with HCM, a maximum thickness of the LV wall of 15–18 mm was found in 62% of patients, and 10 individuals had severe LVH (>20 mm). One patient had an apical aneurysm. Most patients in these subgroups showed myocardial fibrosis (LGE in 77% of patients). TTE GLS was impaired in the patients with HCM LGE(+) compared to the patients with HCM LGE(-), but due to the small number of patients it did not reach statistical significance (−13.3 ± 3.5 vs. 15 ± 3, *p* = 0.6).

Moreover, GLS (optimum value −12.1) revealed predictive values for severe myocardial fibrosis (LGE > 4 LV segments) in the subgroup of HCM with AUC = 0.884 ± 0.49 (95% CI = 0.773–0.953; 95% Bootstrap CI = 0.760–0.960; *p* < 0.001), sensitivity 80% and specificity 83%. This predictive value of GLS was similar both in the HCM subgroup and the study group (HCM + non-HCM).

GLS (optimum value −12.3) was also predictive for moderate fibrosis (LV > 2 segments) in the subgroup of HCM with AUC = 0.725 ± 0.67 (95% CI = 0.591–0.833; *p* < 0.001), sensitivity 50% and specificity 87%. Both AUC values of GLS for LV > 2 segments and >4 segments were similar (*p* = 0.7). However, GLS failed to predict any LGE in patients with HCM (*p* = 0.5).

## 4. Discussion

Our study explored the utility of TTE- and speckle-tracking-derived GLS in patients suspected of HCM, comparing findings with myocardial fibrosis determined by CMR LGE. This was a homogeneous study group with mostly preserved LVEF and free from myocardial pathologies other than LVH. Routine 2D echocardiography established concordance with CMR diagnosis of HCM in 82% of the cases, but it was not reliably associated with the presence or extent of myocardial LGE. In contrast, GLS emerged as the only independent echocardiographic parameter associated with fibrotic burden, showing a moderate linear correlation and a strong association with severe fibrosis (>4 segments). These findings suggest that GLS may provide complementary information beyond conventional TTE parameters; however, these observations should be interpreted cautiously given the sample size and cross-sectional design.

These findings are consistent with previous studies reporting reduced longitudinal strain in myocardial segments with fibrosis, reflecting the sensitivity of STE to subtle alterations in myocardial deformation that precede overt systolic dysfunction [17,19]. In our study, GLS demonstrated only modest discriminatory ability for detecting any myocardial fibrosis (LGE), with relatively low sensitivity at the proposed threshold. Moreover, GLS failed to predict any LGE in the subgroup of HCM. However, its diagnostic performance improved substantially in the identification of more advanced fibrosis (>2 or >4 LV segments), where both sensitivity and specificity were clinically meaningful. This indicates that GLS is more effective in detecting patients with a higher fibrotic burden rather than early or mild myocardial involvement.

From a clinical perspective, GLS should not be considered a replacement for CMR, which remains the reference standard for myocardial tissue characterization and is recommended in current ESC guidelines for patients with suspected HCM. Instead, GLS may serve as an adjunctive imaging parameter associated with myocardial fibrotic burden. However, given the cross-sectional design of our study and the absence of clinical outcome data, its role in guiding referral pathways or prioritizing CMR evaluation remains speculative and requires confirmation in prospective studies. Importantly, due to its limited sensitivity for detecting mild fibrosis, a normal GLS value does not exclude the presence of LGE. On the other hand, GLS demonstrated a high negative predictive value for a significant myocardial fibrosis. It underscores its potential clinical value in refining risk stratification. Given that myocardial fibrosis is a well-recognized substrate for arrhythmias, adverse remodeling, and sudden cardiac death in HCM [20], the ability to identify high-risk patients through echocardiographic strain analysis may facilitate earlier intervention and closer follow-up. The moderate sensitivity of GLS to detect any LGE suggests that it could help to suspect myocardial injury in individual cases that may warrant further evaluation with CMR. However, given the cross-sectional design of our study and the lack of clinical outcome data, no direct conclusions regarding prognostic implications or arrhythmic risk can be drawn. Therefore, the potential role of GLS in risk stratification should be considered hypothesis-generating and requires validation in prospective studies with long-term follow-up.

### 4.1. Echocardiography and GLS

Prior studies showed that impaired strain is associated with myocardial injury and fibrosis in HCM [21,22]. Our observation that GLS was significantly reduced in HCM versus non-HCM subjects and further depressed in cases with LGE aligns with prior studies. It was also demonstrated by Saito et al. that even in HCM with preserved LVEF, GLS was markedly impaired in patients with LGE (−11.8 ± 2.8 in those with LGE vs. −15.0 ± 1.7% without, *p* < 0.001) and independently predicts %LGE on CMR [20]. Another recent study showed that adjusting strain measurement for regional wall thickness improved discrimination of the most hypertrophied segments in HCM (−8.3 ± 3.3% vs. −11.4 ± 4.5%, *p* = 0.002 in fibrotic versus non-fibrotic segments) and enhanced feasibility [21]. In line with our data, strain impairment is increasingly recognized as an early marker of myocardial dysfunction in HCM, preceding volumetric or ejection fraction abnormalities [23]. Importantly, in our cohort GLS exhibited high negative predictive value (95%) for severe fibrosis (LGE > 4 segments) and moderate specificity (87%), although sensitivity was lower (72%). This pattern suggests that while GLS cannot replace CMR for the detection of myocardial fibrosis, it may provide complementary information regarding myocardial involvement. However, its role in clinical decision-making pathways requires prospective validation.

By contrast, conventional indices in our study (LVEF, maximal wall thickness, left atrial size, tissue Doppler E’) did not independently predict fibrotic extent as deformation imaging captures pathological changes earlier than chamber geometry or global systolic metrics. The systematic review and meta-analysis by Yang et al. encompassing 2441 HCM patients found that impaired GLS was significantly associated with major adverse cardiac events (MACE) (HR 1.12 per unit worsening of GLS) and fibrotic burden [24].

### 4.2. Cardiovascular Magnetic Resonance and Myocardial Fibrosis

CMR with LGE remains the non-invasive gold standard for the assessment of myocardial fibrosis, and our findings confirmed its superiority in quantifying fibrotic burden. Importantly, beyond fibrosis quantification, CMR plays a key role in differential diagnosis, as several phenocopies may mimic hypertrophic cardiomyopathy while presenting distinct patterns of myocardial involvement and requiring different clinical management [25].

In our cohort nearly 70% of patients had LGE, with a median of two segments (IQR 0–4) and median total LGE of 2.95% (IQR 0–5.9%). The anatomical predilection for basal anterior and anteroseptal walls mirrors typical HCM patterns [26]. The association between GLS and both the number and severity of LGE-positive segments underscores the complementary value of echocardiographic strain analysis. However, the modest sensitivity of GLS in detecting mild fibrosis highlights that it cannot replace CMR, but rather serves as a screening tool to identify patients warranting advanced imaging.

The observed correlation between GLS and both the number of LGE-positive segments and %LGE (r = 0.42) supports the concept that deformation abnormalities reflect underlying fibrotic substrate. In the landmark ESC guidelines, the presence and extent of LGE are recognized as markers of arrhythmic risk and adverse prognosis in HCM [5]. However, the moderate sensitivity of GLS for mild fibrosis (i.e., smaller numbers of LGE-positive segments) emphasizes that echocardiographic strain should not replace CMR when detailed tissue characterization is required—instead, it can identify those patients for whom CMR is most indicated.

In our ROC analysis, GLS outperformed age and E/E′ in predicting moderate (>2 segments) or severe (>4 segments) fibrosis, highlighting its potential role in guiding imaging strategy. Given constraints in CMR availability in many centers, the incorporation of GLS may help optimize resource utilization.

### 4.3. Myocardial Fibrosis in LVH and Clinical Risk

Myocardial fibrosis in HCM has been implicated in the pathogenesis of ventricular arrhythmias, progressive ventricular dysfunction, heart failure, and sudden cardiac death (SCD) [20]. Numerous studies have established LGE >15% of LV mass as a high-risk feature [26]. Considering that GLS measurement is widely available, non-invasive, and reproducible, its incorporation into standard TTE assessment could enhance risk stratification and follow-up strategies in patients with LVH. In particular, markedly reduced GLS values could indicate a subgroup at higher risk who may benefit from closer surveillance or earlier initiation of targeted therapies.

Furthermore, recent data from the STRAIN-HCM study (*n* = 130, median follow-up 52 months) showed that CMR-feature-tracking-derived GLS was independently predictive of major adverse cardiac events (HR 1.12 per unit, *p* = 0.01) after adjustment for conventional risk factors and LGE [27]. Our results reinforce and extend this evidence into the echocardiographic domain, indicating that GLS measured by TTE may likewise contribute to risk stratification algorithms.

From a clinical perspective, the widespread availability, rapid acquisition, and reproducibility of GLS make it an attractive adjunct to standard TTE in patients with LVH or suspected HCM. In our clinical workflow, markedly reduced GLS (e.g., worse than −12.1% for predicting >4 segments of LGE) might prompt earlier CMR, more frequent follow-up, more aggressive rhythm monitoring, or earlier referral for genetic or therapeutic assessment.

GLS should not be used as a standalone screening tool and should be considered as an adjunctive parameter within a multimodality imaging approach, which is currently regarded as a cornerstone of modern HCM management integrating echocardiography and CMR [7].

## 5. Limitations

This was a single-center study with a moderate sample size, which may limit external validity. Although both speckle-tracking echocardiography and CMR allow for more detailed assessment [11] (e.g., regional strain analysis, T1 or extracellular volume mapping), these were not included in the present analysis. T1 mapping and extracellular volume (ECV) quantification allow the assessment of diffuse myocardial fibrosis, which may not be detected by LGE, and could provide additional insights into the relationship between myocardial deformation and tissue characterization. However, we aimed to assess the utility of a major STE parameter (LV GLS) in relation to a well-evidenced CMR LGE. The cross-sectional study design does not provide prognostic data, including clinical follow-up and holter ECG data. Finally, the GLS cut-offs showed in our ROC analysis are exploratory and should not be used directly in clinical practice to guide management. Larger multicenter studies with longitudinal follow-up are warranted to confirm the prognostic value of GLS in predicting myocardial fibrosis and clinical outcomes in HCM. Genetic testing data were not systematically available in the study population. Therefore, genotype–phenotype relationships and the potential influence of genetic background on myocardial fibrosis and strain parameters could not be evaluated. Although multivariable regression analysis was performed, the relatively moderate sample size and smaller subgroup populations may have limited statistical power and reduced the ability to comprehensively adjust for all clinically relevant confounding factors. To minimize overfitting, the number of variables included in the final model was restricted; however, residual confounding cannot be excluded. Therefore, the observed associations should be interpreted cautiously and considered exploratory rather than definitive. Finally, we did our best to exclude patients with other mechanisms of LVH. However, it is not possible to identify patients with some rare disease leading to LVH and/or fibrosis. Given that recruitment of the study group was focused on the patients with suspected HCM in TTE, our results and conclusions are related to this group of patients instead of general cardiovascular patients (referral bias).

## 6. Conclusions

In patients with LVH and suspected of HCM, GLS derived from standard transthoracic echocardiography showed a significant association with the extent of myocardial fibrosis assessed by CMR. GLS was the only independent echocardiographic parameter associated with myocardial fibrotic burden and demonstrated good discriminatory performance in identifying the patients with extensive LGE. Although GLS cannot replace CMR for detailed tissue characterization, it may provide adjunctive information and may help identify patients with greater myocardial fibrotic burden. The integration of GLS into routine echocardiographic evaluation may provide complementary information regarding myocardial involvement; however, its clinical utility for risk stratification and management decisions requires further prospective validation.

## Figures and Tables

**Table 1:** The clinical characteristics and baseline medical therapy of the study population.

Parameter	Study Group (*n* = 83) Mean ± SD or Median (25–75th Percentile) or Number (%)
**Clinical characteristics**	
Age (years)	57.5 ± 13.3
Female	28 (34%)
NYHA I Class	47 (57%)
NYHA II Class	35 (42%)
HFmrEF	3 (4%)
HFrEF	0 (0%)
Chronic coronary syndrome	27 (32%)
Arterial hypertension class I	35 (42%)
Glomerular filtration rate:	
G1 (≥90 mL/min/1.73 m^2^)	73 (88%)
G2 (60–89 mL/min/1.73 m^2^)	10 (12%)
Diabetes	9 (11%)
Obesity	19 (23%)
Smoking	18 (22%)
**Baseline medical therapy**	
Beta-blockers	83 (100%)
Calcium channel blockers	70(84%)
Anti-arrhythmic drugs	7 (8%)
ACE inhibitors/ARB	47 (57%)
Mineralocorticoid receptor antagonists	5 (6%)
Diuretics	3 (4%)
Statins	58(70%)
**Cardiovascular magnetic resonance**	
LVEDV (mL)	152.2 (48)
LVEF (%)	65 (61–69)
LV mass (g)	157.3 (48.9)
RVEDV (mL)	133 (32)
RVEF (%)	65 (62.1–69)

**Abbreviations**: SD—standard deviation; NYHA—New York Heart Association classification; HFmrEF—heart failure with mildly reduced ejection fraction; HFrEF—heart failure with reduced ejection fraction; ACE—angiotensin-converting enzyme inhibitor; ARB—angiotensin receptor blocker; LVEDV—left ventricular end-diastolic volume; LVEF—left ventricular ejection fraction; LV—left ventricle; RVEDV—right ventricular end-diastolic volume; RVEF—right ventricular ejection fraction.

**Table 2:** Comparison of clinical characteristics of HCM and non-HCM groups.

Parameter	HCM (*n* = 58)	Non HCM (*n* = 25)	
	Mean (SD) or Median (25–75th Percentile)		
**Age (years)**	59.8 ± 11.9	52.4 ± 15.3	*p* = 0.02
**Males**	35 (60%)	20 (80%)	*p* = 0.07
**NYHA I**	24 (41%)	23 (92%)	*p* < 0.001
**NYHA II**	33 (57%)	2 (8%)	*p* < 0.001
**HFmrEF**	3 (5%)	0 (0%)	*p* = 0.25
**HFREF**	n/a	n/a	n/a
**CAD**	20 (35%)	7 (28%)	*p* = 0.53
**Hypertension**	29 (50%)	6 (24%)	*p* = 0.03
**DM2**	6 (10.3%)	3 (12%)	*p* = 0.78
**Obesity**	14 (24%)	5 (20%)	*p* = 0.83
**Nicotinism**	12 (21%)	6 (24%)	*p* = 0.81
**Cardiovascular Magnetic Resonance**			
**LVEDV (mL)**	149 ± 35	158 ± 41	*p* = 0.3
**LVEF (%)**	66 (46–81)	64 (56–74)	*p* = 0.77
**LV mass (g)**	168.6 ± 51	131.3 ± 32	*p* < 0.001
**RVEDV (mL)**	128.1 ± 30.2	144 ± 35.5	*p* = 0.04
**RVEF (%)**	66 (50–69)	65 (54–71)	*p* = 0.7
**Max. LV hypertrophy (mm)**	19.3 ± 4.1	14.1 ± 3.6	*p* < 0.001

Abbreviations: NYHA—New York Heart Association classification of heart failure; HFmrEF—heart failure with moderately reduced ejection fraction; HFrEF—heart failure with reduced ejection fraction; CAD—coronary artery disease; DM2—type 2 diabetes mellitus; SD—standard deviation; LVEDV—end-diastolic left ventricle volume; LVEF—left ventricular ejection fraction; LV—left ventricle; RVEDV—right ventricle end-diastolic volume; RVEF—right ventricle ejection fraction.

**Table 3:** Multivariate linear regression model analysis of echocardiographic parameters and the severity of myocardial fibrosis (LGE) in the patients with LVH.

	LGE%LV				
	β	SE	t	*p*	95% CI
**GLS**	0.66	0.3	2.15	0.03	0.04–1.26
**LVEF**	−0.01	0.10	−0.1	0.91	−0.22–0.19
**Max LVH**	0.41	0.24	1.73	0.08	−0.06–0.89
**LA**	0.16	0.15	1.1	0.27	−0.13–0.45

Abbreviations: GLS—global longitudinal strain; LVEF—left ventricular ejection fraction; LVH—left ventricular hypertrophy; LA—left atrium; TDI E’—tissue Doppler imaging mitral annular early diastolic velocity; 95% CI −95% confidence interval.

**Table 4:** ROC curve analysis of TTE parameters associated with myocardial fibrosis (all *p* < 0.05).

	**LGE in LV**						
**Parameters**	**AUC ± SE**	**Optimum Value**	**Sensitivity (%)**	**Specificity (%)**	**PPV (+) (%)**	**NPV (−) (%)**	**95% CI**
**age (years)**	0.648 ± 67	>55	71	68	83	50	536–750
**E/E’**	0.647 ± 71	>8.3	89	48	80	66	535–749
**GLS (%)**	0.658 ± 63	>−14.3	82	42	57	72	545–758
	**LGE > 2 LV segments**						
**Ultrasound Indexes**	**AUC ± SE**	**Optimum Value**	**Sensitivity (%)**	**Specificity (%)**	**PPV (+) (%)**	**NPV (−) (%)**	**95% CI**
**GLS (%)**	0.775 ± 52	>−15.8	87	52	57	88	671–860
	**LGE > 4 LV segments**						
**Ultrasound Indexes**	**AUC ± SE**	**Optimum Value**	**Sensitivity (%)**	**Specificity (%)**	**PPV (+) (%)**	**NPV (−) (%)**	**95% CI**
**GLS (%)**	0.867 ± 51	>−12.1	72	87	47	95	775–932

Abbreviations: LGE—late gadolinium enhancement; LV—left ventricle; GLS—global longitudinal strain; E/E’—early diastolic to mitral annular early diastolic velocity ratio; AUC—area under the curve; PPV—positive predictive value; NPV—negative predictive value.

## Data Availability

The datasets generated and analyzed during the current study are not publicly available due to patient confidentiality and institutional regulations but are available from the corresponding author on reasonable request.
